# Recalcitrance of *Cannabis sativa* to *de novo* regeneration; a multi-genotype replication study

**DOI:** 10.1371/journal.pone.0235525

**Published:** 2021-08-13

**Authors:** Adrian S. Monthony, Sean T. Kyne, Christopher M. Grainger, Andrew Maxwell P. Jones

**Affiliations:** Department of Plant Agriculture, University of Guelph, Guelph, ON, Canada; SativaGen, UNITED STATES

## Abstract

*Cannabis sativa* is relatively recalcitrant to *de novo* regeneration, but several studies have reported shoot organogenesis or somatic embryogenesis from non-meristematic tissues. Most report infrequent regeneration rates from these tissues, but a landmark publication from 2010 achieved regeneration from leaf explants with a 96% response rate, producing an average of 12.3 shoots per explant in a single drug-type accession. Despite the importance regeneration plays in plant biotechnology and the renewed interest in this crop the aforementioned protocol has not been used in subsequent papers in the decade since it was published, raising concerns over its reproducibility. Here we attempted to replicate this important *Cannabis* regeneration study and expand the original scope of the study by testing it across 10 drug-type *C*. *sativa* genotypes to assess genotypic variation. In our study, callus was induced in all 10 genotypes but callus growth and appearance substantially differed among cultivars, with the most responsive genotype producing 6-fold more callus than the least responsive. The shoot induction medium failed to induce shoot organogenesis in any of the 10 cultivars tested, instead resulting in necrosis of the calli. The findings of this replication study raise concerns about the replicability of existing methods. However, some details of the protocol could not be replicated due to missing details in the original paper and regulatory issues, which could have impacted the outcome. These results highlight the importance of using multiple genotypes in such studies and providing detailed methods to facilitate replication.

## Introduction

Plant tissue culture techniques provide the foundation for advanced biotechnologies such as protoplast regeneration systems, microspore culture for double haploid production, transgenics, genome editing, and other important tools which have yet to be fully explored in *Cannabis sativa* L. Due to prohibition, early micropropagation studies of *Cannabis* were limited in number and scope, with the majority of research relying on a single drug-type genotype or on less regulated industrial hemp cultivars (*Cannabis sativa* with <0.3% w/w tetrahydrocannabinol (THC) in the flowering heads) [1]. These early studies found that both drug-type *Cannabis* (high-THC; >0.3% w/w THC) and industrial hemp can be maintained *in vitro* [2–4] and that plants derived from *in vitro* grown *C*. *sativa* display comparable chemical and physical profiles to greenhouse-grown counterparts [2], with no significant effect on the cannabinoid contents of high-THC genotypes [4,5]. Regulations surrounding the production and consumption of *Cannabis* have since relaxed, creating an increased need and opportunity to explore the application of plant biotechnologies in the *Cannabis* industry. However, without robust and reproducible *in vitro* regeneration systems the widespread application of plant biotechnologies remains unfeasible.

In *C*. *sativa*, the majority of micropropagation studies rely on shoot multiplication from existing meristems found in the apical and axillary buds [6]. Numerous protocols report regeneration from meristematic tissues at levels greater than 70% [4,7–9], with the most effective of these protocols rely on existing meristems found in the nodal tissues of vegetative cuttings. *In vitro* cultures of these tissues can be used to establish multiple shoot cultures (MSC), which have been reported to produce upwards of 9 to 13 shoots from a single nodal segment [4,8]. Unfortunately, most existing protocols are developed using only one high-THC genotype of *C*. *sativa*. Often the most successful methods have used the genotype MX from the University of Mississippi [7,10–12]. Recent evidence suggests that micropropagation protocols developed using a single genotype of *C*. *sativa* may not be replicable when tested on other genotypes, instead resulting in significantly lower multiplication rates [13]. Notably, most micropropagation protocols published in the United States are developed with *Cannabis* supplied by the University of Mississippi, who is funded through the National Institute on Drug Abuse (NIDA) as the sole licensed facility to supply *Cannabis* for research purposes in the United States [14,15]. There is mounting evidence that NIDA supplied *Cannabis* may not be representative of the broader population of *Cannabis* which can be obtained from commercial producers [14–16], and given this apparent discrepancy it is unknown if methods developed using NIDA supplied *Cannabis* will work effectively for commercially relevant genotypes.

Plant biotechnologies such as inter-specific hybridization via protoplast fusion or targeted genome editing using CRISR-CAS9 (clustered regularly interspaced short palindromic repeats- CRISPR associated protein 9) have been well established in many commercially important plant species. However, these technologies have not been successfully applied in *C*. *sativa* due to a lack of reliable and robust regeneration protocols that are required to regenerate plants from somatic cells or protoplasts [17]. In addition to applications in biotechnologies, regeneration from somatic tissues would increase multiplication rates due to the wider range of responsive tissues compared to MSCs, and could be valuable for micropropagation. While most published methods using non-meristematic tissue of *Cannabis* report low levels of regeneration [3,5,18–22], a three-part regeneration system reported by Lata *et al*. [12] has shown success in leaf explants from a high-THC genotype, MX. In this protocol, the authors report optimized callogenesis on Murashige & Skoog (MS) [23] medium with 0.5 μM of α-naphthaleneacetic acid (NAA) and 1.0 μM thidiazuron (TDZ). Regeneration was most successful by transferring cultures to MS medium with 0.5 μM TDZ, resulting in 96.6% of cultures responding with an average of 12.3 shoots per culture [12]. Rooting was subsequently achieved on medium consisting of half-strength MS salts and 2.5 μM indole-3-butyric acid (IBA) [12].

As with many shoot induction methods, this protocol was only tested on MX, but the authors suggest their methodology may be suitable for both male and female plants of any other genotype in the species [12]. Despite the publication of this protocol over a decade ago and the implications it has for advances in *Cannabis* biotechnology, it has not been replicated in subsequent publications by any independent research groups or the original authors. There has been an increasing awareness of what is dubbed the ‘reproducibility crisis’ in scientific literature and over 50% of biologists have reported that they have failed to reproduce published research [24]. This crisis has been attributed to structures in academia that base hiring of faculty, promotion and tenure on the novelty, impact and volume of published literature and to journals who strive to publish “ground-breaking” research often resulting in the refusal to publish validation or replication studies, especially those studies showing negative results [24–26]. The incentivization of novel results arising from clean research narratives has been linked to incomplete reporting of methodologies and the omission of negative results [25] and with a rising number of retractions occurring each year [27] there is a case for more accountability through replication studies and ‘open data’ initiatives. These aforementioned incentives promote novel results with succinct storylines at the expense of robust reporting of methodologies and conducting replication studies. Publication of replication studies is necessary and can prove especially beneficial to emerging fields such as *Cannabis* micropropagation where the body of literature is limited, and existing studies are based on a narrow genetic base from relatively few research groups.

**Fig 1:**
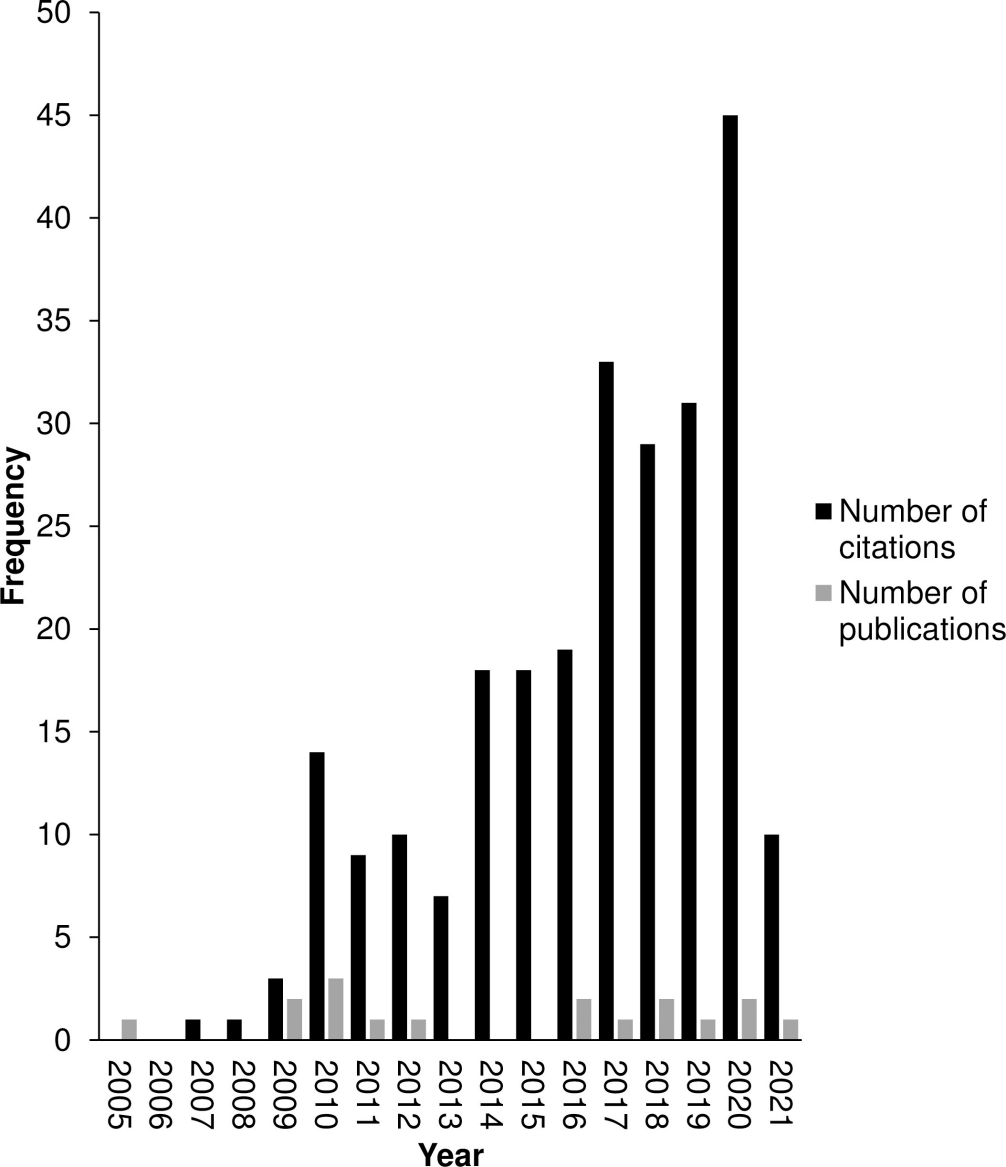
Annual citation frequency and number of new publications on regeneration in *Cannabis sativa*. The annual citation frequency on regeneration in *C*. *sativa* continues to grow, indicating a growing interest in the in vitro regeneration of Cannabis, however new publications on regeneration in *Cannabis* are not keeping pace. Search Topic: “Cannabis sativa” AND “regeneration” AND “in vitro” on the Web of Science database. Data obtained from Web of Science on February 15, 2021 using Microsoft Edge®.

As the regulatory landscape evolves to facilitate research in this nascent field, there is growing interest in *Cannabis* regeneration (Fig 1) and an increasing need for reliable methods. Despite growing interest, the number of drug-type genotypes used in protocol development has historically been very low [5,12,21,28] and only recently have five or more genotypes been included in a single study [13,29]. In stark contrast, regeneration studies on commercial hemp varieties can boast up to 12 genotypes and has identified significant genotypic variability [18]. Generalizing conclusions based on single-genotype studies has faced scrutiny, and this is particularly poignant given the chemical and genetic differences identified between the genotypes used for research purposes and commercially relevant germplasm [14–16]. Despite growing evidence of the diversity of *Cannabis*, there have been limited *in vitro* attempts to assess the effects of this genetic diversity on replicability of existing tissue culture methods [13]. The objective of this study was to replicate the Lata *et al*., 2010 [11] methods as closely as possible across 10 different genotypes to assess the reproducibility of this protocol and to provide insight into the variability in genotypic response.

## Materials & methods

### Regeneration from leaf-derived explants

#### Plant material

All leaf material was sourced from *in vitro* shoots maintained within a long-term *in vitro* germplasm at the University of Guelph. All explants used in this experiment were from Stage 2 *in vitro* grown shoots maintained as previously described [6,13]. The plants had been in a state of vegetative growth for at least 6 months and were routinely subcultured ever 4–6 weeks. The clonal genotypes used in the study (GRC, U22, U31, U37, U38, U42, U61, U82 and U91) were obtained from Hexo Corp. (Gatineau, Quebec) and the heterogenous seed-derived genotype (RTG) was obtained from RotoGrow (Bolton, Ontario). Prior to the experiments all plants had been maintained on semisolid medium composed of DKW nutrients (Phytotechnology Laboratories, KS, USA), 3% sucrose and 6 g/L agar in a controlled atmosphere growth chamber, under a 16-hr photoperiod at 25°C. These plants were maintained in We-V boxes (We Vitro Inc., Guelph, ON) each containing between 8 and 12 female explants. To ensure consistent sampling, leaf explants were taken from young, fully expanded leaves found no lower than 3 nodes below the apical tip of the shoot. The first experiment included two drug-type genotypes: GRC and RTG. After these two genotypes failed to regenerate, the protocol was repeated using a more comprehensive genetic pool of 10 unique genotypes. For the second replication of the experiment, GRC and RTG were selected again, alongside 8 additional clonal genotypes: U22, U31, U37, U38, U42, U61, U82 and U91.

#### Media preparation

The control medium (MS-0) consisted of Murashige & Skoog [23] (MS; M524 Phytotechnology Laboratories, KS, USA) nutrients, 3% sucrose, 0.8% (w/v) type E agar (Sigma Aldrich, St. Louis, MO), and pH adjusted to 5.7 using 1 M NaOH and 1 M HCl [12]. The callus induction medium (hereafter referred to as LT-C) consisted of MS (M524; Phytotechnology Laboratories) nutrients, 3% sucrose, 0.8% (w/v) type E agar (Sigma Aldrich), 0.5 μM NAA (Sigma Aldrich), and 1.0 μM TDZ (Caisson Laboratories, Inc., Smithfield, UT) adjusted to a pH of 5.7 [12]. The shoot induction medium (hereafter referred to as LT-S) consisted of MS (M524; Phytotechnology Laboratories) nutrients, 3% sucrose, 0.8% (w/v) type E agar (Sigma Aldrich), 0.5 μM TDZ (Caisson) and adjusted to a pH of 5.7 [12]. Each glass culture vessel (9.05 cm high × 5.8 cm diameter baby food jars; Phytotechnology Laboratories) contained 25 mL of media which were sterilized by autoclaving for 20 minutes at 121°C and 18 PSIG.

#### Callogenesis

**Fig 2:**
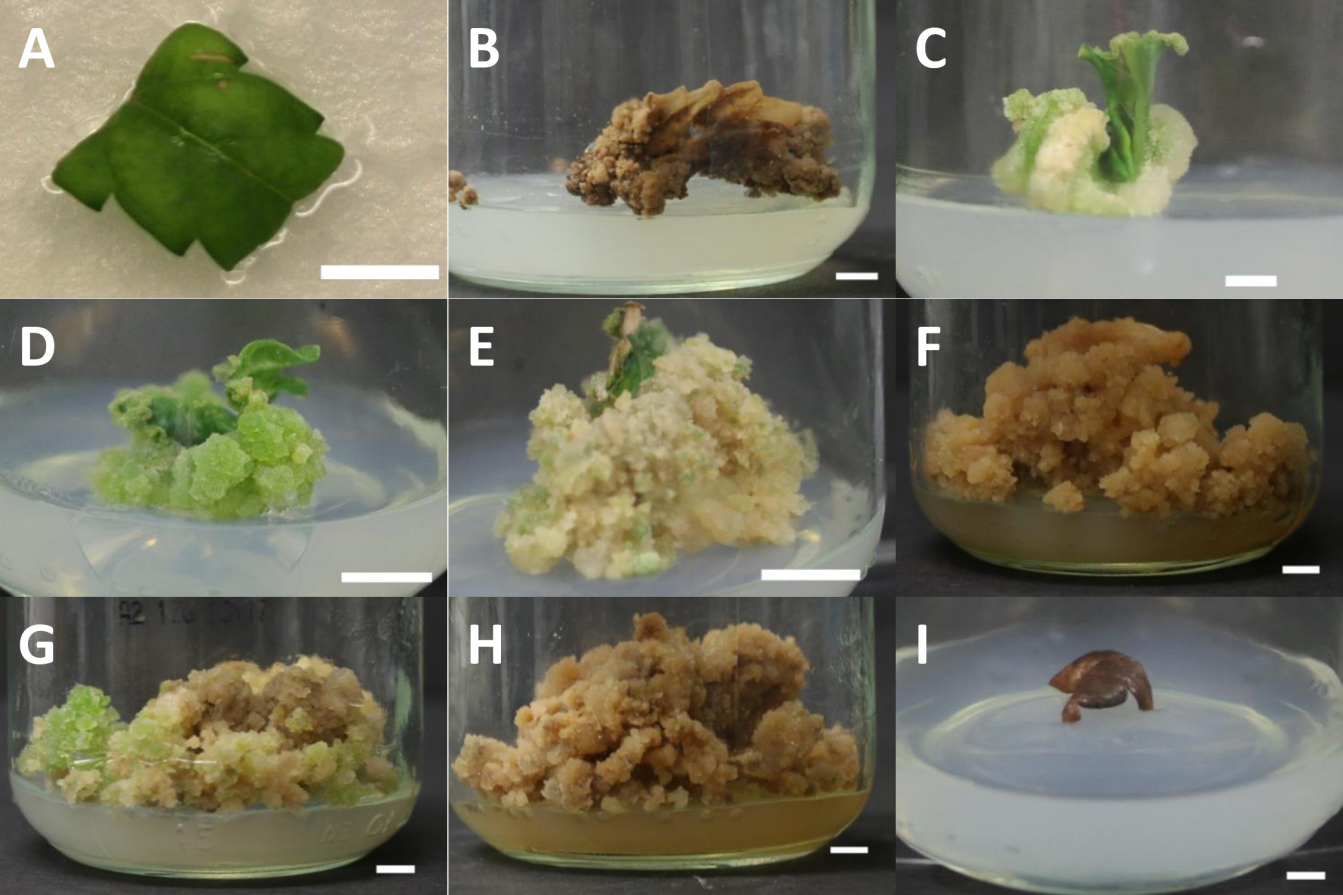
Explant and callus morphology over the course of callus and shoot induction phase of the replication study. A) Representative photograph illustrating the size, colour and portion of the leaf used as leaf explants for callus induction. Midribs but not petioles are included during the dissection of the ~ 1 cm^2^ leaflet. B) Necrotic callus of *C*. *sativa* cv. RTG after 4 months on LT-S medium. C) Cream coloured, friable callus of *C*. *sativa* cv. U91, one month after culture LT-C medium. D) Green, nodular callus of *C*. *sativa* cv. U61, one month after culture LT-C medium. E) Friable callus of *C*. *sativa* cv. RTG, two months after culture on LT-C medium, prior to transfer to shoot induction medium. F) Callus browning in *C*. *sativa* cv. U61 two months after transfer of callus onto LT-S medium. G) Some two-month-old callus on LT-S media showed only slight signs of browning with creamy and green nodular callus still present, pictured: cv. U82. H) Browning was ubiquitous across all cultivars following four months on LT-S medium, as seen by in cv. U82. I) Control leaf explants cultured on MS-0 media failed to callus, as shown by brown and dead leaf explant from cv. GRC. Scale bar in all photographs is 0.5 cm.

Callogenesis was carried out on LT-C media, along with MS-0 as a negative control. Since some details of explant preparation and culture methods were not specified in the original protocol published by Lata *et al*., 2010 [11], decisions were made based on the information provided (See Table 1 for specific comparison of methods). Leaf explants were excised into 1 cm^2^ squares using a sterile scalpel in a biological safety cabinet. All leaf explants were taken along the central axis of the leaflet, so as to include mid-rib tissues (Fig 2A) and excluded petioles. The abaxial side of one explant was carefully placed onto the medium in each glass jar culture vessel and gently tapped down to the surface of the media to maximize contact and mitigate unwanted movement while handling. Ten explants of each genotype were assigned to each treatment (*n = 10*). The capped jars were wrapped twice with Micropore™ tape (3M™, St. Paul, MN), randomized, and placed together in a controlled atmosphere growth chamber, under a 16-hr photoperiod at 25°C. Photosynthetically active radiation (PAR) and light spectral data (S1 Fig) were obtained using an Ocean Optics Flame Spectrometer (Ocean Optics, FL, USA; raw spectral data available on the OSF database: https://osf.io/kdc72/?view_only=5d8879ca2f2e479eb3b7635e1f6e3941). The average PAR of the experimental area was 48.74 ± 3.53 μmol s^-1^ m^-2^. Explants were maintained under these conditions for 8 weeks and checked weekly for callus development. Callus mass and percent response were measured after 8 weeks of culture on LT-C and prior to moving calli to the shoot initiation medium.

**Table 1:** Experimental conditions in the current study compared to Lata *et al*. (2010) [11].

Experimental Condition	Current Study	Lata *et al*. (2010) [11]	Comment
*Plant material and Preparation*			
Genotype	Clonal Genotypes: U22, U31, U37, U38, U42, U61, U82, U91 and GRC Heterogenous (seed derived) Plants: RTG	Clonal Genotype: MX	MX described as a high THC elite female clone [11,12]. See Fig 3. for the genotypes used in the current study.
Explant Source	Young, fully expanded *in vitro* leaves, found no lower than 3 nodes below the shoot apex.	Young *ex vitro* leaves from plants grown in a controlled environment growth room.	Criteria for selecting leaves (location, ages, etc.) N.S. in Lata *et al*. (2010) [11].
Explant Type and Preparation:	Young leaves, cut into squares using sterile disposable scalpels.	Young leaves	Explant preparation not specified in Lata *et al*. (2010). [11]
Explant Size:	100 mm^2^	0.5–10.0 mm	N/A
*Culture Conditions*			
Light Type:	LED	Fluorescent	Type of fluorescent not stated by Lata *et al*. (2010) [11]
Photoperiod:	16 h	16 h	N/A
Light Intensity:	48.74 ± 3.53 μmol s^-1^ m^-2^	~ 52 μmol s^-1^ m^-2^	N/A
Light Spectrum:	See S1 Fig.	N.S.	Light spectra were not provided by Lata *et al*. (2010) [11]
Vessel Type:	5.8 cm × 9.05 cm baby food jars with magenta B caps.	4 cm × 9.5 cm baby food jars with magenta B caps.	N/A
*Media Composition and Preparation*			
Basal Salt:	Murashige and Skoog’s medium (Product ID: M524; Phytotechnology Laboratories)	Murashige and Skoog’s medium (supplier unknown)	N/A
Plant Growth Regulators	0.5 μM NAA (Sigma Aldrich) 1.0 μM TDZ (Caisson Laboratories, Inc.)	0.5 μM NAA 1.0 μM TDZ (Sigma Aldrich)	N/A
Gelling Agent	0.8% (w/v) type E agar (Sigma Aldrich)	0.8% (w/v) type E agar (Sigma Aldrich)	N/A
pH	5.7	5.7	N/A
Sterilization	20 minutes at 121°C and 18 PSIG.	Media was sterilized. (conditions N.S.)	N/A

N.S.- not specified; N/A- not applicable.

* Reported as standard error of the mean.

#### Shoot initiation and rooting

Attempting to initiate shoot organogenesis from the calli, 8-week-old calli cultured on LT-C were moved to baby food jars containing 25 mL of LT-S medium and maintained under the previously described conditions. Control explants from the callogenesis phase (grown on MS-0) which were still living were subcultured to baby food jars containing 25 mL of new MS-0 media following 8 weeks of culture. In the original study by Lata *et al*. (2010) [11], callus cultures were maintained on LT-S until shoots exceeded 2.5 cm in height at which point they were moved to a root induction medium [12]. In the present study, no calli underwent shoot regeneration within the 4 months that they were maintained on LT-S (see Results & Discussion). Calli which had died (Fig 2B; further discussed in the Results & Discussion**)**, or had stopped growing were considered to be non-responsive and destroyed after four months.

### Simple Sequence Repeat (SSR) analysis of cultivars

#### Plant material

Fresh stem tissue obtained from *in vitro* grown explants was used for all DNA extractions. For each clonal genotype (U22, U31, U37, U38, U42, U61, U82, U91 and GRC) three, 1 cm stem segments each obtained from three separate clonal explants were pooled together. Due to the heterozygous nature of the RTG plants, a 2 cm segment was taken from each of the twelve *in vitro* explants used in both experiments and each of these 12 samples were processed separately. The approximate weight of each of these fresh tissue samples was 100 mg.

#### DNA extraction

DNA extraction was performed using NucleoSpin® Plant II Mini (Macherey-Nagel, Dürin, Germany) as per manufacturer’s instructions with minor modifications. The following modifications were made to the manufacturer’s procedure: PL1 and RNase A buffers were added to tissue samples in a 2.0 mL microcentrifuge tube prior to homogenizing. Samples were homogenized using a SPEX SamplePrep 1600MiniG® homogenizer (SPEX, Metuchen, NJ) at 1500 RPM for 1 minute. Following homogenization samples were briefly centrifuged to settle the lysate. Samples were gently resuspended and incubated for 15 minutes at 65°C and extraction was subsequently performed as per manufacturer’s instructions. Extracted DNA was stored at -20°C prior to SSR analysis.

#### SSR genotyping

Purified DNA was thawed at 4°C and gently vortexed prior to quantification. DNA concentration and purity were measured with a NanoDrop ND-1000 spectrophotometer (Thermo Scientific, Waltham, MA). Eleven SSR markers (Table 2) developed by Alghanim & Almirall were used for genotyping [30]. PCR amplification was performed based on the Schuelke method [31]. Each PCR reaction consisted of: 3 μL 20% trehalose, 4.92 μL of molecular-grade H_2_O, 1.8 μL 10X PCR buffer (MgCl_2_), 3 mM dNTP mix, 0.12 μL of 4 μM M13-tailed forward primer, 0.48 μL of 4 μM reverse primer, 0.48 μL of 4 μM “universal” M13 primer labeled with VIC fluorescent dye (Applied Biosystems, Foster City, CA), 0.2 μL of 5.0 U μL^-1^ *taq* polymerase (Sigma Jumpstart™, Sigma-Aldrich, St. Louis, MO), and 3 L of template DNA for a total reaction volume of 15 μL. Amplification reactions were performed using thermocyclers (Eppendorf MasterCycler®, Hauppauge, NY). The conditions of the PCR amplification are as follows: 94°C (5 min), then 30 cycles at 94°C (30 s)/56°C (45 s)/72°C (45 s), followed by 8 cycles at 94°C (30s)/53°C (45S)/72°C (45 s), followed by a final extension at 72°C for 10 minutes. Following the PCR reaction, plates were held at 4°C at the cycle’s end and subsequently stored at -20°C until sequencing. Fragment analysis of the completed PCR products was done using an Applied Biosystems® 3500 Genetic Analyzer (ThermoFisher, Waltham, MA). A dye-labeled size standard (GeneScan 500-LIZ, Life Technologies, Burlington, ON, Canada) was used as the internal size standard, and PCR fragment sizes were determined using a DNA fragment analysis software (GeneMarker, SoftGenetics LLC, State College PA).

**Table 2:** SSR loci and the primers used in this study.

Primer ID	Locus	Repeat	Primers (5’-3’)	Number of alleles	Range of Amplicons (bp)
P4	C11CANN1	(GAT)8(GGT)7	GTGGTGGTGATGATGATAATGGTGAATTGGTTACGATGGCG	7	233–241
P6	B01-CANN1	(GAA)13(A)(GAA)3	TGGAGTCAAATGAAAGGGAAC CCATAGCATTATCCCACTCAAG	11	375–387
P7	D02-CANN2	(CTT)6(ATT)(CTT)10	AGATGATGCCAAGAGGCAC TACCCATCCCCTTGGATCAC	6	120–294
P9	C08-CANN2	(GA)21	GCAAGAAGTAGAGAGACAATCC CCCTCAACACTCATTAACTCAC	10	236–276
P13	H11CANN1	(CT)18	GCATGTGGTTGTTTCGTACCC CAGCGAACATTCACTCTAGCTC	5	349–358
P14	B02-CANN2	(AAG)10	CAACCAAATGAGAATGCAACC TGTTTTCTTCACTGCACCC	7	239–250
P15	H09-CANN2	(GA)15	CGTACAGTGATCGTAGTTGAG ACACATACAGAGAGAGCCC	4	287–292
P17	E07-CANN1	(CTA)9	CAAATGCCACACCACCTTC GTAGGTAGCCAGGTATAGGTAG	5	195–201
P19	B05-CANN1	(TTG)9	TTGATGGTGGTGAAACGGC CCCCAATCTCAATCTCAACCC	6	308–317
P24	D02-CANN1	(GTT)7	GGTTGGGATGTTGTTGTTGTG AGAAATCCAAGGTCCTGATGG	4	197–202
P25	H06-CANN2	(ACG)7	TGGTTTCAGTGGTCCTCTC ACGTGAGTGATGACACGAG	7	332–342

Primers used were first reported by Alghanim & Almirall [30]. Number of alleles and range of amplicon sizes were obtained from experimental results.

#### SSR data processing

**Fig 3:**
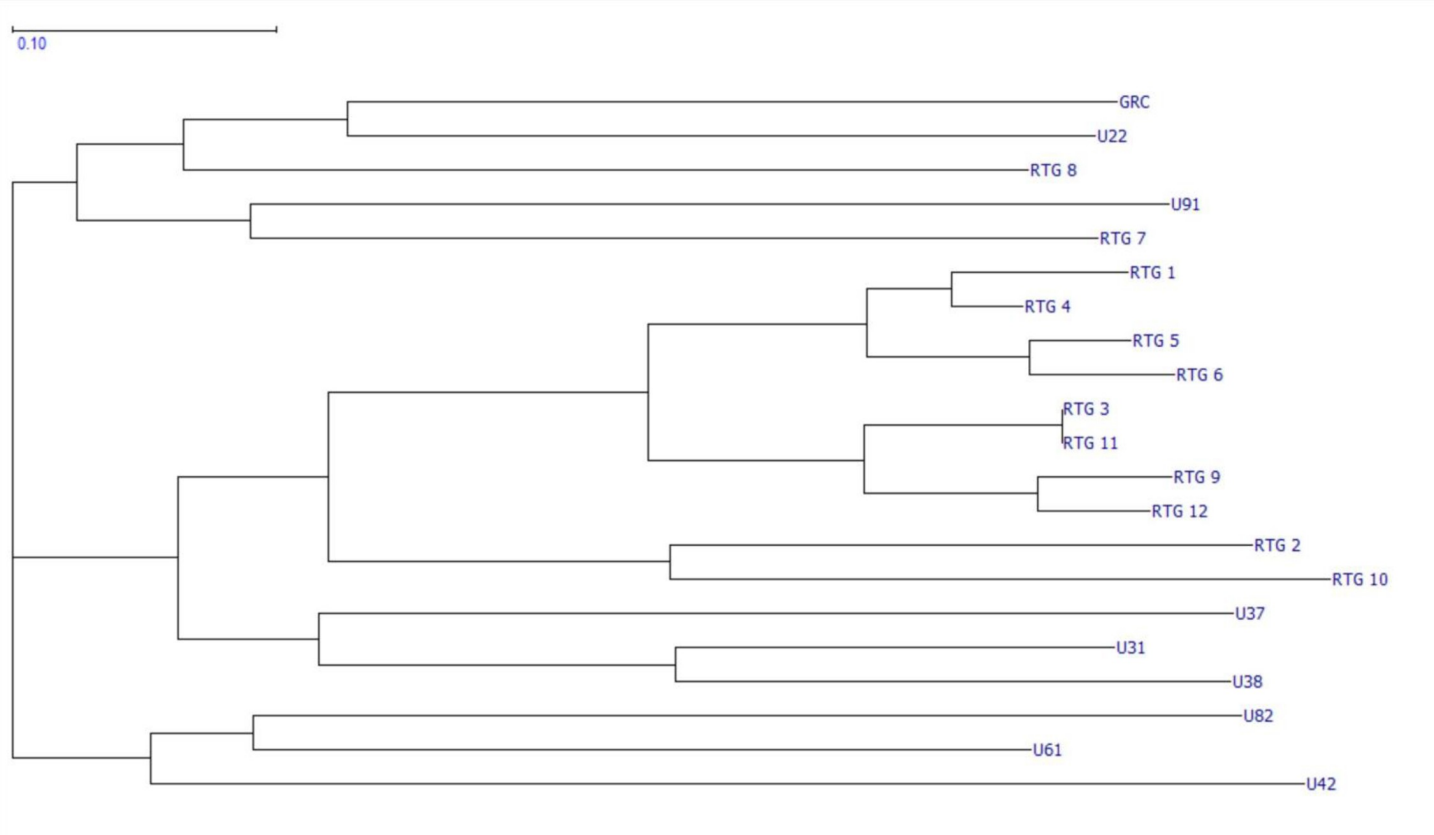
NJ dendrogram depicting relative genetic relatedness across selected cultivars. A visual representation of the relative genetic distance matrix presented as a NJ dendrogram. Data was analyzed using GeneMarker, organized in Excel, and visualized using GGT.

SSR data (available on OSF database: https://osf.io/skd2f/?view_only=2bb948dec29a4a1db3ad4cf7eaece9bb) was analyzed and sorted using GeneMarker (SoftGenetics LLC, State College PA). Results of the SSR allele-call analysis were organized via Microsoft Excel (Microsoft Corp., WA, USA) and imported into GraphicalGenoTyping (GGT 2.0, Wageningen University, NL) and a relative genetic distance matrix was developed to compare pair-wise distance values (available on the OSF database: https://osf.io/skd2f/?view_only=2bb948dec29a4a1db3ad4cf7eaece9bb). This matrix was visually represented as a NJ dendrogram (Fig 3).

### Experimental design and statistical analysis

Regeneration experiments were two-way cross-classified factorials with a completely random design testing two factors: genotype and media. The first experiment was a 2×2 factorial design testing two genotypes (GRC and RTG) against the previously described media MS-0 and LT-C according to the methods laid out by Lata *et al*. [12]. The experiment was replicated as a 10×2 two-way cross-classified factorial with the inclusion of 8 additional genotypes (U22, U31, U37, U38, U42, U61, U82, U91) in addition to GRC and RTG. Each treatment consisted of 10 experimental units (glass culture vessels; *n* = 10), each containing one explant (sampling unit). All statistical analyses were performed using SAS Studio software (v9.4, SAS Institute Inc., Cary, NC, USA). The ANOVA was performed using PROC GLIMMIX and a means comparison of the callus mass of responding treatments was obtained using the LSMEANS statement (α = 0.05). Missing data (due to contaminated cultures) were replaced with ‘.’ in the dataset (available on OSF Database: https://osf.io/94tae/?view_only=c484303807f540b0b94e31779b02253a) and were not processed by PROC GLIMMIX. Multiple comparisons were accounted for by a post-hoc Tukey-Kramer Test. Visual presentation of the SAS data were prepared using Microsoft Excel® (Microsoft Corp., WA, USA).

## Results & discussion

### SSR analysis finds the selected *C*. *Sativa* genotypes are genetically distinct

As shown in (Fig 3), the results of the SSR analysis identify all 10 genotypes as genetically unique. The genotypes are organized into three predominant clusters. As expected, the most closely related samples were found within the seed-derived lineage of RTG-X, however samples RTG-7 and RTG-8 present as outliers and lend support to the argument that *Cannabis* seed is highly heterogeneous. Clustering patterns also suggest genetic relatedness between U3X and RTG-X groups. The information displayed in (Fig 3) demonstrates that the material used in this study represents genetically distinct genotypes of *C*. *sativa*.

### Recalcitrance of *Cannabis* to shoot induction medium

The aim of this study was to replicate the regeneration protocol from leaf derived callus published by Lata *et al*. in 2010 [11] and assess its robustness across multiple genotypes as closely as possible. However, it should be noted that some aspects of the published method could not be accurately replicated due to a lack of detail in the original method and some regulatory restrictions, and these differences could have impacted the outcome. A comprehensive comparison of methods is presented in Table 1. In our hands, callus was successfully induced in all 10 genotypes (Fig 2C–2E), however the subsequent transfer of calli to shoot induction medium failed to initiate shoot organogenesis in any of the 10 tested genotypes (Fig 2F–2H). As such, it can be concluded that either this method is not suitable for inducing *de novo* regeneration across different genotypes, or that one or more of the details that could not be accurately replicated (Table 1) are critical for success. Regardless, these data highlight previous observations that *Cannabis* is a relatively recalcitrant species [6,9,13,22,32] and care must be taken to include multiple genotypes and detailed descriptions of experimental designs to ensure reproducible methods.

When calli developed on LT-C medium was transferred to LT-S medium [12], no shoot organogenesis was observed and instead the calli declined. This was characterized by browning of medium and calli, and death within four months of transfer (Fig 2F–2H). The callus induction rate was 100% across all 10 tested genotypes grown on the LT-C medium (data available on the OSF database: https://osf.io/kevu4/?view_only=1bfc16548f10495d9a20579111a076a0), which is comparable to the callusing rate reported by Lata *et al*. [12]. Callus formation was observed as soon as one week after induction in the earliest genotype, with all genotypes producing callus within two weeks. All genotypes continued to grow callus during the two-month callus induction phase (Fig 2C–2E). No callus formation was observed in any leaf explants in any of the 10 genotypes cultured on the control medium (MS-0) and within 2-month of transfer onto fresh MS-0, all control explants turned brown and died (Fig 2I).

Callusing in the absence of PGRs is uncommon in *Cannabis* and Lata *et al*. (2010) [11] similarly reported no callusing on MS medium without PGRs [12]. Callus formation and appearances have previously been shown to vary across *C*. *sativa* genotypes. Early work from Mandolino & Ranalli [18] looking at the induction of embryogenic callus found that callusing was easily achieved in leaf tissues of 12 hemp genotypes. However, leaf tissues failed to regenerate in most cases, with only hypocotyls from 1 of the 12 tested genotypes regenerating [18]. Ślusarkiewicz-Jarzina *et al*. [3] similarly reported that callogenesis frequency and appearance varied in their screening of explant tissues for organogenic potential in 5 hemp genotypes. They reported that young leaves were most responsive to callusing with 52% of explants responding, however only 1.35% of the calli regenerated [3]. The results of our study confirm that the tested LT-C medium was more effective than previous methods at inducing callogenesis, reaching 100% response across all 10 tested genotypes. This high response rate compared to previous callogenesis studies on hemp could indicate that drug-type genotypes are more responsive to callusing than some industrial hemp genotypes, or that the medium itself is more effective, and future studies to understand how industrial hemp and drug-type genotypes vary in their callogenesis response are warranted.

**Fig 4:**
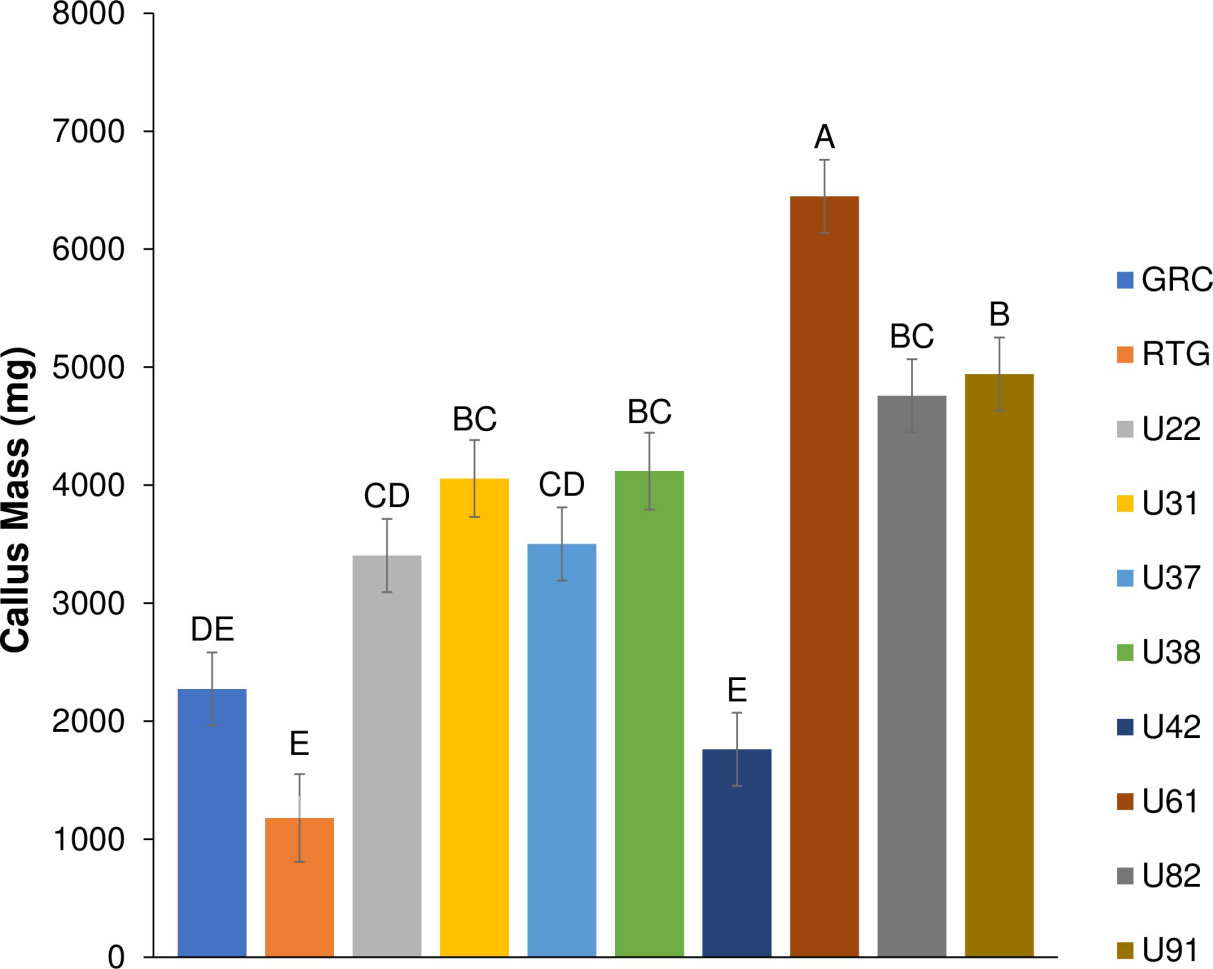
Average callus mass (mg) produced by a 1 cm by 1 cm leaf square following 2 months on LT-C media. All 10 genotypes showed a callus response. Control media contained no PGRs and did not induced callus in any of the genotypes. Same letters indicate that means were not significantly different at p = 0.05 as determined by a Tukey-Kramer multiple comparisons test. Error bars represent standard error.

Little is known about the effect of genotype on callogenesis and regeneration of drug-type *C*. *sativa*. To our knowledge, this study represents the largest attempt to replicate an existing *C*. *sativa* regeneration protocol, and expands upon the original paper’s scope by incorporating 10 SSR-characterized genotypes of *C*. *sativa*. While we found that callusing was obtained on LT-C medium across all 10 tested genotypes, rates of callogenesis were genotype specific (Figs 4 & S2). The average mass of callus differed significantly among genotypes with the most responsive, U61 producing ~5.5-fold more callus than the least responsive genotype, RTG (6448 vs. 1180 milligrams: Fig 4). Callus appearance also varied across genotypes from a friable creamy colour (Fig 2C) to hard nodular green callus (Fig 2D). These differences were apparent within a month of callus induction. Callus growth persisted for the duration of the callus induction phase (2 months; Fig 2E**)**. Our findings that callus formation is not uniform across the genotypes tested suggest that the response is not universal across *C*. *sativa* genotypes, which could also reflect regenerative capacity. Piunno *et al*. [5] tested regeneration potential from three drug-type genotypes and achieved callogenesis and low levels of regeneration in 2/3 of the genotypes, but was unclear from this initial report if this was *de novo* regeneration or proliferation of existing meristems [5]. A subsequently published study showed that shoot proliferation from *C*. *sativa* inflorescences occurs from existing meristematic tissues rather than occurring via *de novo* [33]. A genotype effect was also demonstrated by Wielgus *et al*.(2008) in industrial hemp, who demonstrated that while a callogenesis response existed across the species, the ability to regenerate was genotype specific [20]. These findings were echoed by Chaohua *et al*., who found that the regeneration response was partly genotype dependent [34].

High callusing levels are not uncommon in *Cannabis* despite low to no regeneration. It has been suggested that these high callusing levels are due to elevated levels of endogenous auxins, further supported by the strong apical dominance and rooting capacity of the species [8]. Regeneration of explants from somatic tissue requires the right auxin: cytokinin ratio. High endogenous levels of auxin may contribute to callus production across the10 tested genotypes, but no regeneration occurred; however, further research is required to evaluate this hypothesis The variability in cultivars response to MS media with TDZ for callogenesis and regeneration was recently demonstrated by Chaohua *et al*. [34] whose callogenesis and shoot induction protocol used MS medium supplemented with TDZ and NAA for both callogenesis and shoot induction, rather than separate callogenesis and shoot induction media as proposed by Lata *et al*. [12]. Chaohua *et al*. reported this media reduced the shoot induction period reported by Lata *et al*. from 2 months to 4 weeks, however rates of regeneration were considerably lower [34]. Work by Page *et al*. [13] has shown that the use of DKW basal salts with TDZ [35] may promote increased levels of callogenesis in some *Cannabis* genotypes, suggesting that the interaction between media composition and genotype may affect the formation of callus and subsequent regeneration potential. Smýkalová *et al*. [8] and Wróbel *et al*. [9] have also suggested that frequent subculture is required in order to maintain the regenerative potential of callus cultures of *Cannabis*; however, as no mention of subculturing during callus or shoot induction phases was made in the author’s original work, they were not performed in this replication study [12].

Within 2 months of culture onto the LT-S medium calli began producing phenolic compounds in the media (Fig 2F), a response indicative of stress. It is important to note that a smaller number of explants showed a plateau in growth and no signs of stress (Fig 2G). By 4 months on the LT-S media, most calli showed signs of necrosis and phenolic production (Fig 2H), while those not producing phenolics were considered to be non-responsive to the shoot induction treatment as replicated from Lata *et al*. [12] and were destroyed. We suggest that frequent subculturing, as suggested by Smýkalová *et al*. [8] and Wróbel *et al*. [9] may have reduced the stress of cultures and helped promote regeneration in this study. While outside of the scope of this replication study, we suggest that future *C*. *sativa* regeneration studies assess the effect of subculturing on regeneration from somatic tissues.

### Limitations of this study

The hallmark of a reproducible protocol is the thorough and complete reporting of the materials and methods. As has already been mentioned, efforts were made to replicate the conditions described by Lata *et al*. (2010) [12], however; a number of critical details were omitted in the original publication, making a complete replication of the study impossible. These included the frequency of subculture (if any) during the stages of growth, light spectra used, growth conditions of the source plants, and details on explant preparation (Table 1). Light spectra have been shown to play an important role in the induction regeneration from calli in many species [36–39] and inclusion of spectral information is important to the accurate replication of a study on the induction of explant regeneration from somatic tissues (raw spectral data used in this study may be found on the OSF database at: https://osf.io/kdc72/?view_only=5d8879ca2f2e479eb3b7635e1f6e3941). Tissue type plays an important role in regeneration and the methods presented in the 2010 protocol did not include sufficient details about the preparation of the leaf explants: neglecting to specify which tissue types, such as mid-ribs or petioles, were included in leaf explants; how the leaf explants were excised, and what position within canopy of the source plant they were collected from. As with any multi-lab replication, there are conditions which cannot be accounted for and this is particularly true for heavily regulated plants such as *Cannabis*. For example, the MX genotype from NIDA and the University of Mississippi used by Lata *et al*. (2010) was omitted in this study, as it is not commercially available. Regardless of this omission, the authors of the 2010 study suggest that their methods were robust and should work for *Cannabis* in general [12]. However, this suggestion is inconsistent with emerging evidence from genetic, morphological and chemical studies of *Cannabis* showing considerable variation within commercially available *Cannabis* populations [15,16,40,41]. Furthermore, recent evicence has highlighted that NIDA supplied genotypes are chemically and genetically distinct from many commercially available drug-type *Cannabis* genotypes [14–16]. Together, these factors raise questions about the applicability of this protocol in commercially relevant drug-type genotypes.

Another potential cause for the observed recalcitrance could be the use of *in vitro* grown leaves rather than leaves collected from a controlled environment setting [12]. *In vitro* grown leaves were used in the present study due to legal restrictions preventing the use of greenhouse leaves in our research facilities at the time. This discrepancy could also contribute to the recalcitrance, but further research is needed. The outcome of this replication study may have also been influenced by what *in vitro* plant researchers have described anecdotally as “the local effect”, wherein *in vitro* plant growth and responses vary between locations, lab environments or seasons despite the fact that they are maintained *in vitro* under controlled conditions. These local effects are speculated to be responsible for reducing the efficacy of well established methods when replicated in different locations. Although care was taken in the current study to closely replicate the the methods set out by Lata *et al*. (2010) [11] it is possible that any one, or several of the differences we have highlighted (Table 1) could be critical for success and that regeneration could be achieved if they were addressed. As such, the lack of reproducibility found here could be a result of minor differences in our methodology instead of, or along with genotypic variability.

Despite these differences, we highlight that the protocol is difficult to reproduce and suggest that many genotypes of *C*. *sativa* may show recalcitrance to existing regeneration protocols. Recalcitrance of select genotypes to *in vitro* regeneration has been well documented across multiple agronomically important crops such as cotton, cereals and legumes and many studies have attempted to elucidate the underlying cause [42–44]. Despite decades of research, the factors involved in genotype specific recalcitrance in some species still elude researchers, forcing breeders to rely on backcrossing with more competent genotypes for the introduction of traits into recalcitrant cultivars of high agronomic value. As the development of future *C*. *sativa* regeneration protocols continues, incorporation of multiple genotypes is paramount to understanding the scope of recalcitrance in the species and potentially identifying responsive genotypes for breeding purposes.

## Conclusion

This replication study is the first independent validation of the methods put forth by Lata *et al*. (2010) [11] and expands beyond the scope of the original study by testing the method’s replicability across 10 genetically unique drug-type *Cannabis* genotypes. We show that the most successful callus induction medium (MS with 1.0 μM TDZ+0.5 μM NAA) proposed by Lata *et al*. effectively induced callus growth in all 10 tested drug-type *Cannabis* genotypes, although the callus growth and quantity was found to be species-specific. Despite the successful induction of callus using their 2010 method, we were unable to successfully initiate regeneration by transferring callus to MS medium supplemented with 0.5 μM TDZ in any of the 10 tested genotypes. The failure of this part of the method, which originally reported regeneration levels exceeding 96%, raises doubts about the original authors’ claims that this protocol can be used on any genotype of the species. These findings suggest that regeneration of *Cannabis* from somatic tissues is highly genotype specific. As new evidence emerges showing the genetic and chemical nonuniformity of the species, we suggest that the development of methods meant for the entire genus using single genotypes may no longer represent a viable path forward in *Cannabis* tissue culture.

## Supporting information

S1 Fig Light spectrum used for *in vitro* growth of plant materials. A representative light spectrum of the lighting used in the controlled environment growth chamber. The average photosynthetically active radiation (PAR) over the experimental area was 48.74 ± 3.53 μmol s^-1^ m^-2^ using an OceanOptics. Average PAR was calculated using Excel ™. Full raw spectral data is available at: https://osf.io/kdc72/?view_only=5d8879ca2f2e479eb3b7635e1f6e3941.
(TIF)

S2 Fig Average callus mass (mg) produced by a 1 cm by 1 cm leaf square following 2 months on LT-C media in genotypes GRC and RTG. Callogenesis was first tested in two commercially available genotypes prior to a subsequent screening of the complete 10 genotypes. Callogenesis was achieved on MS media supplemented with 1.0 μM TDZ and 0.5 μM NAA. Control media contained no PGRs and did not induced callus in any of the tested. Same letters indicate that means were not significantly different at p = 0.05 as determined by a Tukey-Kramer multiple comparisons test.
(TIF)
