# Supplementary figures and images for: Recalcitrance of Cannabis sativa to de novo regeneration; a multi-genotype replication study
Source: PLoS One. 2021 Aug 13;16(8):e0235525. doi: 10.1371/journal.pone.0235525 (PMC8363012; doi:10.1371/journal.pone.0235525)

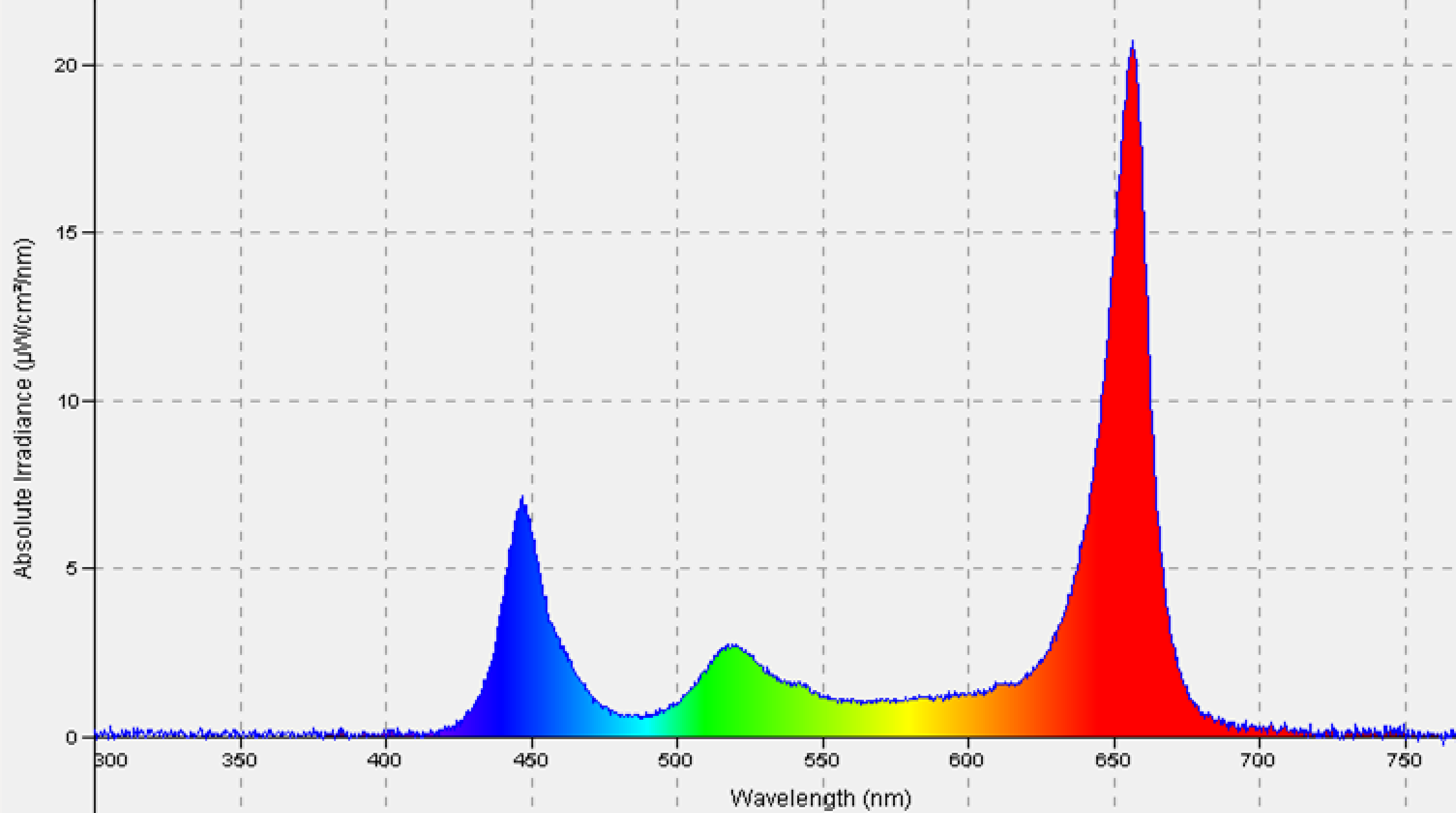

Supplement: S1 Fig — A representative light spectrum of the lighting used in the controlled environment growth chamber. The average photosynthetically active radiation (PAR) over the experimental area was 48.74 ± 3.53 μmol s-1 m-2 using an OceanOptics. Average PAR was calculated using Excel ™. Full raw spectral data is available at: https://osf.io/kdc72/?view_only=5d8879ca2f2e479eb3b7635e1f6e3941. (TIF) [file pone.0235525.s001.tif]

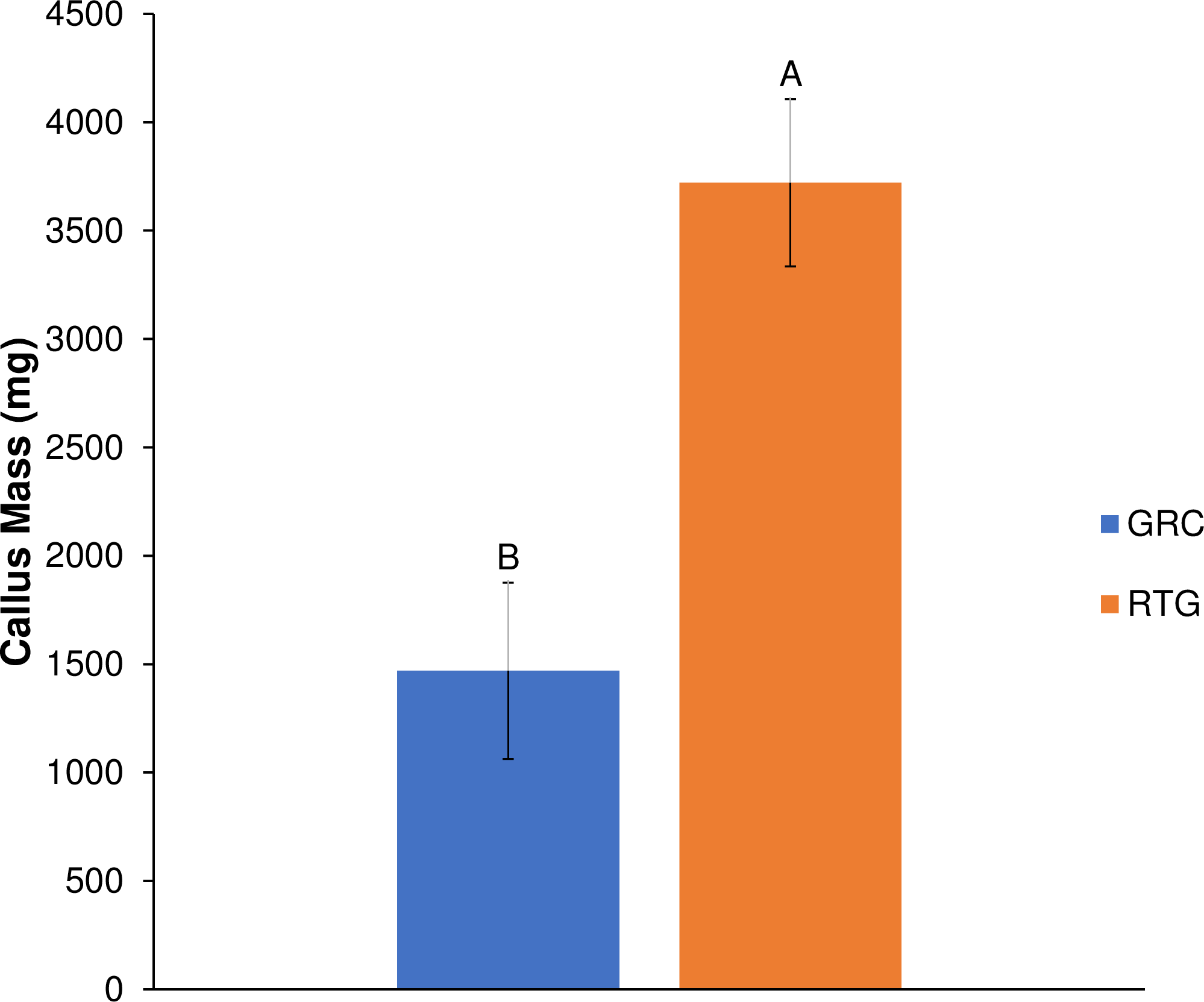

Supplement: S2 Fig — Callogenesis was first tested in two commercially available genotypes prior to a subsequent screening of the complete 10 genotypes. Callogenesis was achieved on MS media supplemented with 1.0 μM TDZ and 0.5 μM NAA. Control media contained no PGRs and did not induced callus in any of the tested. Same letters indicate that means were not significantly different at p = 0.05 as determined by a Tukey-Kramer multiple comparisons test. (TIF) [file pone.0235525.s002.tif]
